# Systemic inflammation in the pathogenesis of irritable bowel syndrome associated with obesity

**DOI:** 10.25122/jml-2021-0120

**Published:** 2021

**Authors:** Yuliya Vyacheslavivna Bilooka, Olexander Ivanovich Fediv, Hanna Yaroslavivna Stupnytska, Vyacheslav Vasilievich Bilookyi, Yurii Yevgenovych Rogovyi, Michael Ivanovich Sheremet, Valentin Nicolae Varlas, Oleksandr Vyacheslavovich Bilookyi

**Affiliations:** 1. Department of Internal Medicine and Infectious Diseases, Bukovinian State Medical University, Chernivtsi, Ukraine; 2. Department of Pathologic Physiology, Bukovinian State Medical University, Chernivtsi, Ukraine; 3. Surgery Department No.1, Bukovinian State Medical University, Chernivtsi, Ukraine; 4. Department of Obstetrics and Gynaecology, Carol Davila University of Medicine and Pharmacy, Bucharest, Romania

**Keywords:** irritable bowel syndrome, obesity, cytokines, endotoxicosis, oxidative stress, CP – ceruloplasmin, CRP – C-reactive protein, IL-10 – interleukin-10, IP – 8-isoprostane, IBS – irritable bowel syndrome, TFNα – tumor necrosis factor-α, TGFβ1 – transforming growth factor-β1

## Abstract

Investigation of the mechanisms promoting the development of irritable bowel syndrome (IBS) in obese patients is one of the most important issues of modern medicine. We examined 97 patients suffering from IBS. The group of comparison included 10 individuals with obesity. The control group included 21 practically healthy individuals. The levels of C-reactive protein (CRP) in the blood serum, tumor necrosis factor-α (TFNα), transforming growth factor-β1 (TGFβ1), interleukin-10 (IL-10), 8-isoprostane (IP), ceruloplasmin (CP) were examined. Endotoxicosis intensity was identified by the content of average molecular peptides in the blood and the Limulus Amebocyte Lysate (LAL) test. In the case of IBS with prevailing diarrhea, especially its comorbid course with obesity, cytokine imbalance was observed, which was manifested by a decreased amount of IL-10 in the blood serum and increased levels of TNFα and TGFβ1. Patients suffering from irritable bowel syndrome with prevailing diarrhea associated with obesity were characterized by high levels of C-reactive protein, fibrinogen and average molecules, increased content of pro-inflammatory cytokines (TFNα and TGFβ1) with a decreased content of IL-10, as well as imbalance of the pro-oxidant and anti-oxidant blood systems (increased content of 8-isoprostane and ceruloplasmin).

## Introduction

Irritable bowel syndrome (IBS) is one of the most spread functional disorders of the gastrointestinal tract, making up an important medical-social issue since it affects an individual's quality of life and social activity [1, 2].

A chronic course of irritable bowel syndrome, polymorphism of clinical signs, and the risk of transformation into organic pathology determine a concept of low-intensity systemic inflammation as one of the mechanisms of IBS pathogenesis [3–6]. IBS is associated with increased content of pro-inflammatory cytokines in the blood, which correlates with the severity of clinical signs of the disease and intensity of extra-intestinal symptoms [7].

Obesity is considered as a condition of chronic subacute systemic inflammation which is associated with an increased synthesis of pro-inflammatory cytokines by both adipocytes and macrophages of the adipose tissue (interleukin (IL)-1β, tumor necrosis factor-α (TFNα), CRP). Moreover, the higher the IP intensity is, the bigger the number of macrophages migrating into the adipose tissue (especially in the visceral one) is [8].

Considering the facts mentioned above, investigating the mechanisms of a comorbid course of IBS and obesity is relevant and timely.Our objective was to study systemic inflammation and oxidative stress indices in patients with IBS and associated obesity with prevailing diarrhea or constipation.

## Material and Methods

Ninety-seven patients with IBS (37 men and 60 women aged 22 to 56 years) were examined and divided into four groups: group I – IBS with diarrhea (18 people), Group II – IBS with constipation (19 people), group III – IBS with diarrhea in combination with obesity (30 people), group IV – IBS with constipation in combination with obesity (30 people). At the same time, studies were conducted on 10 obese patients (comparison group) and 21 practically healthy individuals. Verification of the diagnosis of IBS was performed according to existing criteria [9].

The level of C-reactive protein (CRP), tumor necrosis factor-α (TNFα), transforming growth factor-β1 (TGFβ1), interleukin-10 (IL-10), 8-isoprostane in the blood was determined by enzyme-linked immunosorbent assay (ELISA). Oxidative stress was assessed by the concentration of 8-isoprostane using ELISA. The ceruloplasmin (CP) content in the blood serum was also determined by Revin’s method. The intensity of endotoxicosis was determined by the level of medium molecular peptides in the blood using the method of NI Gabrielyan and the Limulus Amebocyte Lysate (LAL) test. Statistical processing of the obtained data was performed using Biostat 2009 Professional, version 5.8.4.3 (AnalystSoft Inc.).

## Results

Clinical characteristics of patients are shown in Table 1.

**Table 1.** Clinical characteristic of patients.

**Parameters**	**PHI (control) n=21**	**Obesity (group of comparison) n=10**	**IBS + diarrhea (I group) n=18**	**IBS + constipation (II group) n=19**	**IBS + diarrhea +obesity (III group) n=30**	**IBS + constipation + obesity (IV group) n=30**
**Age**	32.81±1.36	32.00±2.19	38.56±2.59	35.56±2.27	35.53±1.81	34.2±1.89
**Sex**	14/7	7/3	12/6	11/8	18/12	19/11
**BMI**	22.19±0.37	31.1±1.04	22.28±0.53	24.56±0.64	32.10±0.37	32.10±0.33
**Duration of the disease**	-	5.1±0.99	4.94±0.36	4.78±0.38	5.07±0.32	4.87±0.34

BMI – body mass index; IBS - irritable bowel syndrome; PHI - practically healthy individuals.

Analysis of the level of pro-inflammatory and anti-inflammatory cytokines in the serum showed the presence of cytokine imbalance in patients with IBS and obesity, both with the predominance of diarrhea and constipation (Table 2). However, in patients with a predominance of diarrhea, we found significantly more pronounced changes, particularly a 15% lower level of IL-10. At the same time, this parameter was lower than that of practically healthy individuals by 31.6%, 25.1%, 34.8% and 23.3% in the comparison group, group I, group III and group IV, respectively. However, in group III, it was lower by 24.3% compared to group II.

**Table 2.** Parameters of systemic inflammation, pro- and antioxidant blood systems in patients with IBS associated with obesity.

**Parameters**	PHI (control) n=21	Obesity (group of comparison) n=10	IBS with diarrhea (I group) n=18	IBS with constipation n=19 (II group)	IBS with diarrhea. Obesity (III group) n=30	IBS with constipation + obesity (IV group) n=30
**IL-10.**	9.90±0.59	6.77±0.31 p<0.05	7.42±0.37 p<0.05 p1>0.05	8.59±0.29 p>0.05 p1>0.05 p2>0.05	6.45±0.25 p<0.05 p1>0.05 p2>0.05 p3<0.05	7.59±0.36 p<0.05 p1>0.05 p2>0.05 p3>0.05 p4<0.05
**TNFα.**	7.57±0.19	16.98±1.80 p<0.05	27.23±1.03 p<0.05 p1<0.05	24.73±1.11 p<0.05 p1<0.05 p2>0.05	35.11±1.26 p<0.05 p1<0.05 p2<0.05 p3<0.05	29.97±1.08 p<0.05 p1<0.05 p2>0.05 p3<0.05 p4<0.05
**TGFβ1.** **pg/ml**	33.37±0.43	37.58±0.86 p<0.05	43.27±0.76 p<0.05 p1<0.05	36.51±0.67 p<0.05 p1>0.05 p2<0.05	47.28±1.02 p<0.05 p1<0.05 p2<0.05 p3<0.05	41.85±0.77 p<0.05 p1<0.05 p2>0.05 p3<0.05 p4<0.05
**CRP.** **mg/l**	3.67±0.38	7.10±0.90 p>0.05	11.56±1.36 p<0.05 p1<0.05	7.53±0.76 p<0.05 p1>0.05 p2<0.05	14.00±1.08 p<0.05 p1<0.05 p2>0.05 p3<0.05	9.19±0.90 p<0.05 p1>0.05 p2>0.05 p3>0.05 p4<0.05
**Fibrinogen.** **g/l**	3.30±0.09	4.39±0.16 p<0.05	4.33±0.16 p<0.05 p1>0.05	3.59±0.27 p>0.05 p1>0.05 p2>0.05	4.61±0.26 p<0.05 p1>0.05 p2<0.05	3.47±0.19 p>0.05 p1<0.05 p2<0.05 p3>0.05 p4<0.05
**8-isoprostan.** **ng/ml**	1.29±0.02	1.46±0.07 p>0.05	2.01±0.09 p<0.05 p1<0.05	1.51±0.04 p>0.05 p1>0.05 p2<0.05	2.30±0.09 p<0.05 p1<0.05 p2<0.05 p3<0.05	1.64±0.06 p<0.05 p1>0.05 p2<0.05 p3>0.05 p4<0.05
**Ceruloplasmin.** **mg/l**	0.92±0.02	1.33±0.11 p<0.05	1.89±0.08 p<0.05 p1<0.05	1.61±0.08 p<0.05 p1>0.05 p2<0.05	2.02±0.07 p<0.05 p1<0.05 p2>0.05 p3<0.05	1.64±0.06 p<0.05 p1<0.05 p2<0.05 p3>0.05 p4<0.05
**Average molecules.**	0.234±0.002	0.249±0.004 p<0.05	0.250±0.005 p<0.05 p1>0.05	0.244±0.002 p<0.05 p1>0.05 p2>0.05	0.257±0.015 p<0.05 p1>0.05 p2>0.05 p3<0.05	0.248±0.002 p<0.05 p1>0.05 p2>0.05 p3>0.05 p4<0.05

BMI – body mass index; CRP – C-reactive protein; IBS – irritable bowel syndrome; IL-10 – interleukin-10; TGFβ1 – transforming growth factor-β1; TNF-α – tumor necrosis factor-α.

Pro-inflammatory cytokines such as TNFα and TGFβ1 were significantly higher by 17.2% and 13.0% in patients of group III compared to patients of group IV, respectively. The level of TNFα was higher in obese patients (2.2 times), in patients of group I (3.6 times), in patients of group II (3.3 times), in patients of group III (4.6 times), and in patients of group IV (4.0 times) than in practically healthy individuals. The highest concentration of TGFβ1 was observed in the group of patients with IBS with diarrhea and obesity (41.7% higher than in almost healthy individuals; 29.5% higher than in patients with diarrhea without obesity). In addition, in groups I and IV, its level was significantly higher by 15.6% and 14.6%, respectively, than in group II.

The level of CRP was higher than in practically healthy individuals: 3.1 times in group I, 2.1 times in group II, 3.8 times in group III, and 2.5 times in group IV. It was found that in group I, it was 34.9% higher than in the presence of constipation; in group III, it was higher by 24.7% and 52.3% than in groups II and IV, respectively. The fibrinogen content in cases of obesity, IBS with diarrhea, IBS with obesity and diarrhea exceeded the corresponding values in almost healthy individuals by 33.0%, 31.2%, and 39.7%, respectively. In patients of group III, it was 28.4% higher than in group I and 32.9% higher than in group IV.

When studying the primary marker of oxidative stress, namely 8-isoprostane, it was found that its level in the serum of patients of group III was 1.8 times higher than control, 1.6 times than the comparison group; 14.4% – group I, 1.5 times – group II and 40.2% – group IV. In groups I and IV of patients, its value was also higher than the control group by 55.8% and 27.1%, respectively. In patients with IBS without obesity, the content of 8-isoprostane was higher in group I compared with group II (by 24.9%).

The level of ceruloplasmin was also higher in group III patients (2.2 times compared with the group of practically healthy individuals, 51.9% compared with obese patients, 25.5% compared with group II and 23.2% compared with group IV). In obese patients, it was 44.6% higher, in group I, it was 2.1 times higher, in groups II and IV 1.8 times higher than in PZO. With the benefits of diarrhea, the ceruloplasmin level was 17.4% higher than with the benefits of constipation.

When studying the rate of endogenous intoxication, namely the content of medium molecules in blood plasma, a similar trend with ceruloplasmin was found; however, it was less pronounced.

Thus, in IBS with diarrhea, especially in its comorbid course with obesity, there is an imbalance of cytokines, which was manifested through a reduced content of IL-10 with increasing levels of TNFα and TGFβ1.

The results of our study correlate with the data indicating that in the case of IBS, especially with diarrhea, cytokine imbalance occurs by an increase of pro-inflammatory and decrease of anti-inflammatory ones [10]. Many other pieces of research demonstrated the role of cytokines in IBS pathogenesis and their association with the manifestation of clinical signs [11, 12]. IBS is known to be a multi-factorial disease. It was associated with functional disorders only before. However, the participation of the immune system and the development of low-grade chronic inflammatory syndrome with this pathology has been evidenced. The role of systemic inflammation with obesity, especially with its abdominal type, is aslo demonstrated [13, 14].

## Discussion

Thus, it could be suggested that IBS with prevailing diarrhea stipulates inflammatory changes not only in the intestines but at the systemic level, and comorbid obesity intensifies the chronic systemic inflammatory process. We found out that patients with IBS, prevailing diarrhea and comorbid obesity develop a higher intensity of systemic inflammation, which was characterized by a high level of CRP in the blood serum and fibrinogen level. Oxidative stress is confirmed to be one of the main parts of pathogenesis in the case of many diseases, including gastrointestinal pathology [15–21]. Numerous studies demonstrated an imbalance of the pro- and antioxidant blood systems with chronic gastrointestinal tract diseases and IBS in particular [22–27].

Thus, we found an imbalance of the pro- and antioxidant blood system in IBS with diarrhea and obesity, as evidenced by high levels of 8-isoprostane and ceruloplasmin. The level of medium molecules in the blood plasma showed that the level of endogenous intoxication is more pronounced in IBS with diarrhea and obesity.

## Conclusion

Patients with irritable bowel syndrome with diarrhea and obesity are characterized by an increased content of C-reactive protein, fibrinogen, and medium molecules, pro-inflammatory cytokines (TNFα and TGFβ1) with a decreased content of IL-10, as well as an imbalance of prooxidant and antioxidant systems (an increased content of 8-isoprostane and ceruloplasmin).

## Acknowledgments

### Ethical approval

The approval for this study was obtained from the Ethics Committee of the Bukovinian State Medical University and Chernivtsi Regional Hospital, Ukraine (approval ID: 11-02/11/2019).

### Consent to participate

The participants gave written informed consent for participation in this study.

### Conflict of interest

The authors declare that there is no conflict of interest.
